# Inferior Vena Cava Syndrome

**DOI:** 10.14309/crj.0000000000002057

**Published:** 2026-03-27

**Authors:** Anandu Mathews Anto, George Sarin Zacharia, Rayan Faisal Grib Alataa, Dmitry Lvovsky

**Affiliations:** 1 Department of Pulmonary Medicine, BronxCare Health System, New York, NY; 2 Department of Internal Medicine, BronxCare Health System, New York, NY

**Keywords:** inferior vena cava, ascites, cirrhosis, hepatic vena cava syndrome, obstructive shock

## Abstract

Inferior vena cava syndrome (IVCS) is an infrequent but potentially life-threatening condition resulting from obstruction of the inferior vena cava (IVC), leading to impaired venous return. Hepatic vena cava syndrome is a rare cause of IVCS. We report the case of a 58-year old man with a complex medical and surgical history who presented with abdominal pain and distension. He had cirrhosis of the liver with moderate ascites, and diagnostic paracentesis confirmed spontaneous bacterial peritonitis. The patient developed refractory hypotension requiring vasopressors, encephalopathy, and hypoxic respiratory failure necessitating mechanical ventilation. Imaging revealed severe focal stenosis of the subdiaphragmatic IVC with preserved hepatic and portal venous patency, raising concern for IVCS. The patient underwent interventional radiology–guided percutaneous transluminal balloon angioplasty of the stenotic segment of the IVC, resulting in prompt improvement in hemodynamic status. This case highlights IVCS as an under-recognized cause of obstructive shock and underscores the importance of prompt recognition and endovascular intervention to avert morbidity and mortality in this subset of patients.

## INTRODUCTION

Inferior vena cava syndrome (IVCS) is a clinical entity caused by obstruction of the inferior vena cava (IVC). It could result from intraluminal causes like thrombosis, extrinsic compression, or tumor infiltration.^1^ Hepatic vena cava syndrome (HVCS) is a less frequent cause of IVCS in the Western world, but is reported more frequently across Asia and Africa, and is one of the leading causes of cirrhosis and ascites in Nepal.^2^ Irrespective of the etiology, IVCS can severely impede venous return to the heart, leading to a reduction in cardiac preload and potentially contributing to a state of obstructive shock.^3^ This impairment of venous flow is reflected in the portal circulation, leading to portal hypertension and, subsequently, ascites. The syndrome could be particularly detrimental in patients with preexisting cardiopulmonary or liver diseases. We report a case of IVCS, most probably due to HVCS, successfully managed with interventional radiology-guided transvenous dilatation of the involved segment of IVC.

## CASE REPORT

A 58-year-old Vietnamese man with a history of chronic obstructive pulmonary disease, pulmonary hypertension, seizure disorder, heart failure with preserved ejection fraction, and ethanol use disorder, presented to the emergency department with progressively worsening, diffuse abdominal pain radiating to the back of 3 days' duration. He reported concomitant nausea and multiple episodes of nonbilious, nonbloody vomiting and reduced bowel movements.

His history was eventful with cecal perforation and peritonitis in 2018, requiring right hemicolectomy and diverting ileostomy. Postoperatively, the patient developed wound dehiscence, wound infection, and adhesive small intestinal obstruction, requiring re-exploration, adhesiolysis, wound debridement, and closure with retention sutures. After a protracted hospital course of nearly 2 months, the patient was readmitted thrice in the subsequent 6 months with small bowel obstruction, presumed adhesive, and treated conservatively. The stoma was ultimately reversed in 2020.

On admission, the patient was tachycardic, seemed anxious, and exhibited tremors on extended arms, but denied having hallucinations. Physical examination was significant for diffuse abdominal distension and tenderness, with shifting dullness. Initial laboratory evaluation revealed anemia, thrombocytopenia, and a deranged hepatic profile (Table 1). The patient was initiated on intravenous fluids, thiamine supplements, and chlordiazepoxide for presumed alcohol withdrawal syndrome. However, his clinical condition worsened rapidly, and he became hypotensive, requiring vasopressors, and was admitted to the intensive care unit.

**Table 1.** Summary of the laboratory workup of the patient

Laboratory test	Results			Normal range
	Day 0	Day 3	Day 7	
Total leukocyte count	14.7	10	6.3	4.8–10.8 k cells/mm^3^
Hemoglobin	10.5	9.3	9.4	12–16 g/dL
Platelet count	129	110	89	150–400 k cells/mm^3^
Sodium	133	142	145	135–145 mEq/L
Potassium	3.5	3.4	3.5	3.5–5 mEq/L
Blood urea nitrogen	8	6	11	6–20 mg/dL
Creatinine	0.5	0.5	0.6	0.5–1.5 mg/dL
Total protein	4.4	5.7	—	5.8–8.3 g/dL
Albumin	1.7	3	—	3.2–4.6 g/dL
Total bilirubin	3.7	3.4	—	0.2–1.1 mg/dL
Direct bilirubin	2	2.1	—	0–0.3 mg/dL
Aspartate aminotransferase	32	47	—	9–36 unit/L
Alanine aminotransferase	48	51	—	5–40 unit/L
Alkaline phosphatase	143	158	—	30–130 unit/L

Chest x-ray revealed hyperinflation without any acute cardiopulmonary processes. An emergent noncontrast computed tomography (CT) of the abdomen demonstrated a cirrhotic liver and voluminous ascites. No hepatic mass lesion was identified; the gall bladder, biliary tree, and pancreas were normal. A diagnostic paracentesis confirmed a high serum–ascites albumin gradient and low-protein ascites, with a polymorphonuclear count of 3,560 cells/mm^3^ (Table 2), consistent with portal hypertension-related ascites and spontaneous bacterial peritonitis (SBP). Samples were sent for peritoneal fluid culture, and he was initiated on intravenous piperacillin–tazobactam. However, the patient continued to require vasopressors and developed encephalopathy and hypoxic respiratory failure, requiring endotracheal intubation and mechanical ventilation.

**Table 2.** Ascitic fluid analysis

Laboratory test	Paracentesis 1	Paracentesis 2
Total leukocyte count (cells/mm^3^)	4,000	875
Polymorphonuclear count (cells/mm^3^)	3,560	700
Total protein (g/dL)	0.6	—
Albumin (g/dL)	0.2	—
Serum–ascites albumin gradient	1.5	—
Lactate dehydrogenase (U/L)	113	—
Glucose (mg/dL)	76	—
Amylase (U/L)	3	—

**Figure 1.**
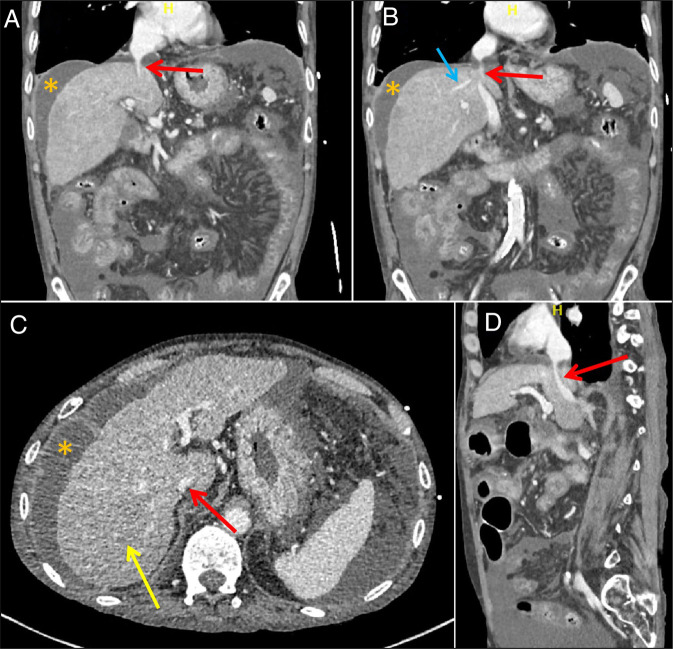
Contrast-enhanced computed tomography images, venous phase. (A and B) Coronal images, revealing IVC stricture (red arrow), ascites (yellow star), and patent hepatic vein (blue arrow). (C) Axial image demonstrating the narrowed IVC (red arrow), ascites (yellow star), and heterogeneous, nodular liver consistent with cirrhosis (yellow arrow). (D) Sagittal projection showing stenotic IVC (red arrow). IVC, inferior vena cava.

A bedside point-of-care ultrasound revealed hyperdynamic ventricles with apparently small chambers and a markedly small IVC. Further evaluation with abdominal ultrasound with Doppler demonstrated moderate ascites and a markedly narrowed IVC, measuring approximately 8 mm in diameter, with patent hepatic and portal veins. The constellation of clinical and radiological findings was concerning for IVC stenosis, with reduced preload and obstructive shock. The patient underwent contrast CT abdomen, which revealed a stenotic subdiaphragmatic hepatic IVC (Figure 1). Subsequently, a conventional IVC venography was performed through the right femoral vein; it demonstrated a patent but severely stenotic subdiaphragmatic IVC. Percutaneous balloon angioplasty was performed using sequential balloon dilation by the interventional radiology team. Postintervention venography showed marked improvement in IVC caliber and venous return. In addition, the patient demonstrated improvement in hemodynamic status and reduced vasopressor requirements after the intervention.

A repeat ascitic fluid analysis at 48 hours of initiation of antibiotics revealed excellent response (Table 2). Large-volume paracentesis was performed to decompress the abdominal cavity, supplemented with albumin infusions. The ascitic fluid and blood cultures failed to identify a specific pathogen. He was tapered off vasopressors and subsequently liberated from the mechanical ventilator. Intravenous antibiotics were continued for a total of 7 days. He was discharged on ciprofloxacin prophylaxis for secondary prevention of SBP. He is planned for outpatient follow-up and upper gastrointestinal endoscopy. In case of recurrence of IVC stenosis, the interventional radiology team is also exploring the feasibility of IVC stenting.

## DISCUSSION

IVCS is a rare clinical phenomenon characterized by IVC luminal narrowing because of intravascular, intramural, or extravascular causes; it compromises venous return and increases upstream venous pressure. The causes include bland or tumor thrombosis of the IVC or IVC compression by intra-abdominal neoplasia. Congenital anomalies of the IVC, such as membranous obstruction, webs, and May-Thurner syndrome, as well as IVC filters and Broviac hyperalimentation catheters, are also associated with IVCS.^1,4^ Case reports of localized ascites compressing the IVC have been reported in the literature.^5^ A lesser-known cause is HVCS, characterized by narrowing of the IVC at the level of hepatic venous inflow. The inciting event is presumed to be localized bacterial thrombophlebitis of the IVC, which resolves with fibrosis, stricture, and stenosis.^6,7^ The exact mechanism by which the subdiaphragmatic, hepatic segment of IVC is specifically involved in septic thrombophlebitis is unclear. It is postulated that the constant diaphragmatic movement and turbulence of flow due to near-perpendicular drainage of hepatic veins into the IVC in this region make this region relatively more vulnerable to endothelial injury and phlebitis.^2,6^

Our patient had cirrhosis, portal hypertension, ascites, and SBP in the absence of any overt malignancy. He had features of obstructive shock, possibly related to IVCS, as evidenced by the initial low cardiac chamber volumes and narrow IVC, and subsequent dramatic hemodynamic improvement with IVC dilatation. Overall, there was little, if any, concern regarding the diagnosis of IVCS, but what caused it was more intriguing. No IVC intraluminal lesion was identified on CT, Doppler, or venogram; nor were there any infiltrating or compressing neoplasia. In the setting of tense ascites, high intra-abdominal pressures can lead to IVC compression, reduced cardiac preload, and compromised splanchnic circulation.^8^ Tokai et al reported a case of loculated ascites in a liver transplant patient, presenting as IVCS.^5^ Our patient had no tense or loculated ascites to account for IVC compression; generalized ascites would not explain focal IVC stenosis. Alternate causes of postsinusoidal portal hypertension were also considered in the list of differentials in this patient. Sinusoidal obstruction syndrome or veno-occlusive disease of the liver is unlikely; the patient lacks the risk factors for the same, and the syndrome should not lead to a narrow IVC. Constrictive pericarditis or chronic right heart failure is expected to be associated with a dilated IVC, in contrast to the narrow IVC demonstrated in this case. Budd-Chiari syndrome is characterized by intraluminal occlusion of the hepatic veins, which may extend to the IVC.^9^

Our patient, an immigrant from South Asia with a history of intra-abdominal infections and multiple abdominal surgeries, has a severe, stenotic subdiaphragmatic IVC without any intraluminal occlusion or extraluminal compression, and has features most consistent with HVCS. This rare syndrome often runs an indolent course, with exacerbations typically precipitated by infections and presenting as abdominal pain, ascites, and a deranged hepatic function panel.^2^ However, as in our patient with IVCS, it can contribute to a picture of refractory shock. The patient's persistent tachycardia and hypotension, despite vasopressor support, were likely compounded by the severe limitation in cardiac preload from the vena cava luminal compromise. Patients with concomitant cardiopulmonary diseases may not be able to mount an adequate response to the increased afterload and decreased preload imposed by the ascites and elevated intra-abdominal pressure. In addition, the elevated diaphragm, increased pressure transmitted across it, and pleural effusions further impede right atrial filling.

The diagnosis of IVCS is often suspected by imaging, but requires careful interpretation. In patients with cirrhosis and ascites, the IVC tends to be smaller than in other patients, partly because of extrinsic pressure and partly due to reduced net intravascular volume. The definitive diagnostic maneuver is an IVC venogram, performed using either conventional or magnetic resonance techniques.^1,2,10^ The basic principle of IVCS management is to relieve the occlusion. Percutaneous transluminal angioplasty with or without stenting offers prompt relief of occlusion.^2^ Thrombotic occlusions will require thrombolysis or anticoagulation.^11^ Patients with HVCS should receive antibiotics because the disease itself and its exacerbations are closely associated with infections.^6^ In patients with tense ascites, large-volume paracentesis lowers the intra-abdominal pressure.^12^

IVC syndrome is a rare but critical clinical entity characterized by occlusion of venous flow through the IVC, resulting in upstream venous congestion. Involvement of the posthepatic IVC leads to portal hypertension, ascites, and cirrhosis, when chronic. HVCS is a lesser-known cause of IVC syndrome, mostly reported from Asia and Africa. This case report highlights the need to maintain a high index of suspicion regarding this rare entity even in the developed world because prompt diagnosis and interventions could mitigate the morbidity and mortality associated with this rare syndrome.

## DISCLOSURES

Author contributions: RFG Alataa, AM Anto and GS Zacharia conceptualized, wrote, edited, and reviewed the manuscript. GS Zacharia and RFG Alataa performed the literature review and curated the data. AM Anto, RFG Alataa, and D. Lvovsky performed the investigation. D. Lvovsky provided supervision. AM Anto is the article guarantor.

Financial disclosure: None to report.

Informed consent was obtained for this case report.

## ABBREVIATIONS:

CT: computed tomography
HVCS: hepatic vena cava syndrome
IVC: inferior vena cava
IVCS: inferior vena cava syndrome
SBP: spontaneous bacterial peritonitis
